# Acute phlegmonous esophagitis presenting as chest pain: A case report

**DOI:** 10.1097/MD.0000000000036364

**Published:** 2023-12-01

**Authors:** Mose Chun, Daesup Lee, Mun Ki Min, Ji Ho Ryu, Min Jee Lee

**Affiliations:** a Department of Emergency Medicine, Pusan National University Yangsan Hospital, Yangsan, South Korea.

**Keywords:** Acute phlegmonous esophagitis, case report, chest pain, digestive disease, dysphagia, emergency department

## Abstract

**Rationale:**

Acute phlegmonous esophagitis (APE) is bacterial infection of the submucosal and muscularis layers of the esophagus. APE is a rare but life-threatening disease, and few studies have reported it.

**Patient concerns:**

A 63-year-old Korean woman was admitted to the emergency department complaining of chest pain. Contrast-enhanced computed tomography revealed diffuse esophageal wall thickening with low attenuation and paraesophageal fluid collection in the mediastinum. Esophagomyotomy, mediastinal abscess drainage with a right thoracotomy, and left 3-port video-assisted thoracoscopy were performed in the operating room.

**Diagnoses:**

Contrast-enhanced computed tomography revealed diffuse esophageal wall thickening with low attenuation and paraesophageal fluid collection in the mediastinum.

**Interventions:**

Esophagomyotomy, mediastinal abscess drainage with a right thoracotomy, and left 3-port video-assisted thoracoscopy were performed in the operating room.

**Outcomes:**

The patient followed up through an outpatient visit 4 days later discharged. The patient progress was good, and she decided to visit the patient if she had pain afterwards.

**Lessons:**

As APE is rare but deadly, strategies to identify APE in patients with chest pain or dysphagia are needed in emergency department.

## 1. Introduction

The causes of chest pain range from benign and self-limiting to serious and life-threatening (such as unstable angina, aortic dissection, and pulmonary embolism). The differential diagnosis for patients presenting to the emergency department with chest pain should include acute myocardial infarction, aortic dissection, pericarditis, and pulmonary embolism.^[1]^ Acute phlegmonous esophagitis (APE) is an uncommon, but life-threatening, condition characterized by sudden chest pain.^[2]^ This condition should be differentiated from routine cases of chest pain diagnosed in the emergency department.

APE is diffuse inflammation of the esophagus.^[3]^ It is associated with a high mortality rate and causes esophageal stenosis or perforation, mediastinitis, and emphysema.^[2]^ Phlegmonous infections involving the stomach are the most common, while esophageal infections have rarely been reported.^[4]^ This report presents a case of APE recently encountered in our emergency department. This study is expected to aid in the differential diagnosis of patients with chest pain who present to the emergency department.

## 2. Case presentation

A 63-year-old Korean woman was admitted to the emergency department complaining of chest pain. The patient had a 2-day history of difficulty swallowing fluid due to odynophagia. She complained of squeezing pain extending from her sternum to her neck that started the night prior to her presentation. Her chest pain was accompanied by shortness of breath and cold sweats. The patient had a history of hypertension and experienced cerebral infarction 4 years prior to her presentation, and no history of smoking or alcohol consumption. She had no history of diabetes or dyslipidemia. The patient had a blood pressure of 130/90mHg, heart rate of 96 beats/min, oxygen saturation of 98% on room air, and body temperature of 36.7°C. The patient had elevated C-reactive protein (18.16 mg/dL) with leukocytosis (white blood cell count: 13.70 × 10E3/μL; neutrophil count: 12.18 × 10^3^/μL). Her cardiac profile was within the normal range (Troponin I: <0.01 ng/mL; creatine kinase-myocardial band: 1.2 ng/mL). She had hyperglycemia (glucose: 402 mg/dL; glycated hemoglobin: 12.8%), suggestive of uncontrolled diabetes.

**Figure 1.**
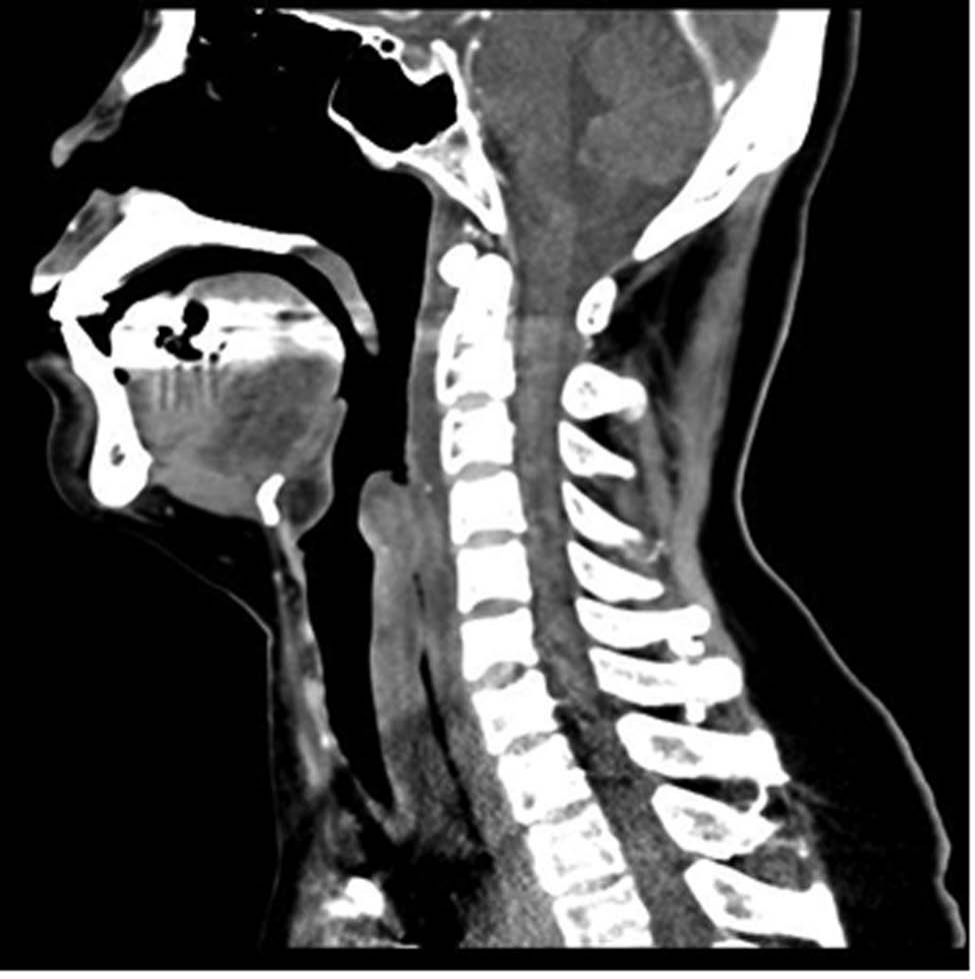
Contrast-enhanced chest computed tomography of a sagittal view reveal diffuse esophageal wall thickening with low attenuation and paraesophageal fluid collection in the mediastinum along the entire length of the esophagus.

A chest contrast-enhanced computed tomography (CT) image revealed diffuse esophageal wall thickening with low attenuation and paraesophageal fluid collection in the mediastinum along the entire length of the esophagus (Fig. 1), suggesting APE and adjacent mediastinitis with suspicious mediastinal abscess formation.

The Department of Gastroenterology was consulted for internal drainage using an endoscope. However, due to diffuse wall thickening of the entire esophagus, an endoscopic approach was not recommended based on the risk of perforation and mediastinitis. Based on the patient history and evaluation results, she was diagnosed with APE.

## 3. Treatment

The patient was subsequently referred to the Department of Cardiothoracic Surgery for surgical treatment. Esophagomyotomy, mediastinal abscess drainage with a right thoracotomy, and left 3-port video-assisted thoracoscopy were performed in the operating room. The patient was administered ciprofloxacin (200 mg, bid, intravenous) for 4 weeks. After several pleural effusion biopsies, *Acinetobacter baumannii* was detected.

**Figure 2.**
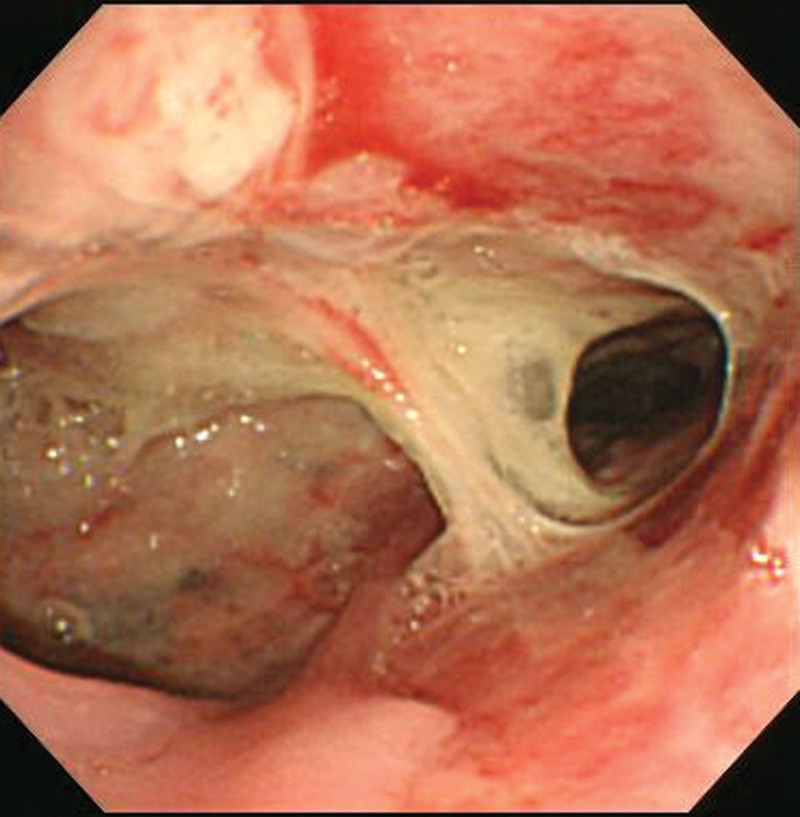
Esophagogastroduodenoscopy reveals pus in the mucosal and submucosal layers and esophageal stricture.

Esophagography was performed 2 weeks after the patient presentation, revealing narrowing of the distal esophagus (Fig. 2). However, the contrast agent passed from the esophagus to the stomach in a normal manner. One week later, a swallowing test revealed aspiration. A percutaneous endoscopic gastrostomy was performed to prevent aspiration pneumonia. Endoscopic gastrotomy and Bougienage were performed to treat esophageal stenosis, and a feeding tube was inserted. The patient was discharged 1 day after the feeding tube was removed and treated with proton pump inhibitors on an outpatient basis. The study was approved by the Institutional Review Board of Pusan National University Yangsan Hospital (no. 05-2021-276).

## 4. Discussion

Phlegmon refers to acute inflammation. Phlegmonous esophagitis can affect the gastrointestinal tract via a diffuse inflammatory process.^[5]^ Predisposing factors include an immunocompromised status, alcoholism, diabetes, peptic ulcer disease, and other injuries to the gastric mucosa.^[5]^ These risk factors cause damage to the gastrointestinal tract, making it vulnerable to bacterial infections.^[6]^ The patient in this report had never been diagnosed with diabetes, though a high glycated hemoglobin discovered in the emergency room indicated new-onset diabetes. Based on this risk factor, it was assumed that the patient had developed APE.

Previously-reported cases of APE began with pain in the pharynx, retrosternal region, or epigastric region. Some patients with APE complain of dyspnea.^[3]^ A previous study reported that the time from symptom onset to APE treatment ranged from 2 days to 3 weeks, with a median of 5 days.^[4]^ The patient in this report had a 2-day history of difficulty swallowing and a 1-day history of chest pain; the time to treatment was relatively short in this patient.

APE is difficult to diagnose in emergency departments as emergency diseases, such as acute myocardial infarction, aortic dissection, or pulmonary embolism, must first be ruled out in patients presenting to the emergency department with chest pain.^[1]^ As APE is a rare disease, it is difficult to differentiate based on pathological signs or symptoms. However, after excluding these emergency conditions, the possibility of acute esophagitis should be considered. On endoscopy, general narrowing of the lumen, difficulty in expansion, and ulcers are typically observed in patients with APE. The most useful diagnostic modality is CT, which demonstrates diffuse thickening of the esophageal wall.^[2,5]^ APE is characterized by hypodense circumferential findings on contrast-enhanced CT. Contrast-enhanced CT occasionally reveals air bubbles in the esophageal wall, which thickens due to infection with gas-forming pathogens.^[6]^ Rapid access to CT is important for an accurate diagnosis of APE and the determination of the need for surgical intervention. Treatment of APE includes the administration of systemic antibiotics, prevention of contamination, provision of nutritional support, preservation of digestive tract continuity, and timely surgical intervention, when necessary. Surgical resection of the esophagus is required when APE is accompanied by esophageal necrosis, progressive esophageal stricture, gastric mucosal atrophy, or acute peritonitis.^[7]^ In the present case, the patient had an esophageal stricture; therefore, esophagomyotomy and mediastinal abscess drainage were performed via video-assisted thoracoscopy.

## 5. Conclusion

In summary, this study reports a rare case of APE in a woman who presented to the emergency room with chest pain. APE is a rare, but highly lethal, disease. Strategies of identifying APE in patients with chest pain or dysphagia are needed.

## Author contributions

**Writing – original draft:** Mose Chun.

**Writing – review & editing:** Mun Ki Min, Ji Ho Ryu, Min Jee Lee, Daesup Lee.
